# The growth of methylammonium lead iodide perovskites by close space vapor transport

**DOI:** 10.1039/d0ra01640c

**Published:** 2020-04-23

**Authors:** Alexander J. Harding, Austin G. Kuba, Brian E. McCandless, Ujjwal K. Das, Kevin D. Dobson, Babatunde A. Ogunnaike, William N. Shafarman

**Affiliations:** Institute of Energy Conversion, University of Delaware Newark Delaware 19716 USA wns@udel.edu +1-302-831-6200; Materials Science and Engineering, University of Delaware Newark DE 19716 USA; Chemical and Biomolecular Engineering, University of Delaware Newark DE 19716 USA

## Abstract

Vapor deposition processes have shown promise for high-quality perovskite solar cells with potential pathways for scale-up to large area manufacturing. Here, we present a sequential close space vapor transport process to deposit CH_3_NH_3_PbI_3_ (MAPI) perovskite thin films by depositing a layer of PbI_2_ then reacting it with CH_3_NH_3_I (MAI) vapor. We find that, at *T* = 100 °C and pressure = 9 torr, a ∼225 nm-thick PbI_2_ film requires ≥125 minutes in MAI vapor to form a fully-reacted MAPI film. Raising the temperature to 160 °C increases the rate of reaction, such that MAPI forms within 15 minutes, but with reduced surface coverage. The reaction kinetics can be approximated as roughly first-order with respect to PbI_2_, though there is evidence for a more complicated functional relation. Perovskite films reacted at 100 °C for 150 minutes were fabricated into solar cells with an SLG/ITO/CdS/MAPI/Spiro-OMeTAD/Au structure, and a device efficiency of 12.1% was achieved. These results validate the close space vapor transport process and serve as an advance toward scaled-up, vapor-phase perovskite manufacturing through continuous vapor transport deposition.

## Abstract 2

This is the first demonstration of an all-vapor close space vapor transport process to deposit methylammonium lead iodide perovskites.

## Graphical abstract

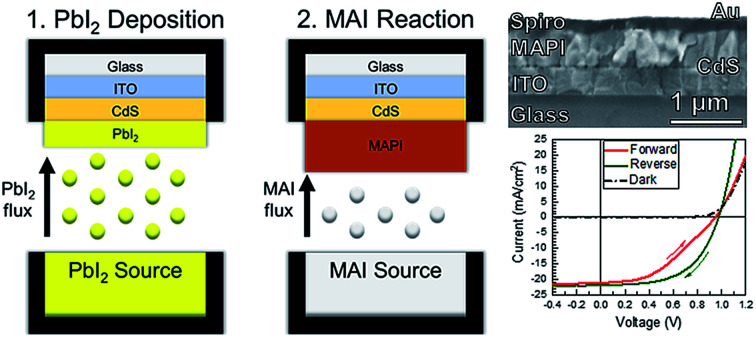

## 1 Introduction

As the stability^1,2^ and efficiency^3–6^ of lead halide perovskite solar cells improve, scaling laboratory performance to industrial production becomes increasingly important. The majority of published research currently focuses on scaling established perovskite spin-coating processes to continuous solution processes such as slot-die coating or blade-coating.^7–11^ However, high-efficiency perovskites can be fabricated using a variety of vapor deposition techniques with established pathways to solvent-free, scaled-up manufacturing.^12–15^ One such method is “close space vapor transport” (CSVT), which serves as a laboratory precursor for large-area, commercial production through vapor transport deposition (VTD).^16^ In CSVT, the source material of interest is evaporated into a carrier gas, transported to a substrate, and condensed to form a thin film. The technique provides insight into how different temperature and pressure regimes, in various carrier-gas environments, can be used to control the deposition rate, uniformity, and crystallinity of the deposited films before designing and implementing scaled-up VTD reactors. CSVT has been used extensively for CdTe solar cell research, where it was regularly used to produce high-quality films that resulted in high-efficiency solar cells.^16^ This fostered the deployment of a commercial VTD process for large-area, uniform CdTe deposition by First Solar LLC.^16^

Despite this success, an all-vapor CSVT process for perovskite thin film deposition has not been investigated. Therefore, feasible processing temperatures and pressures have not been defined, and their effect on deposition rate, film quality, and device performance are unknown. There are only a few reports of methylammonium iodide (CH_3_NH_3_I, MAI) reactions in a CSVT configuration with spin-coated PbI_2_ followed by annealing to drive a reaction between PbI_2_ and MAI vapor.^17–19^ However, these studies do not provide sufficient information to develop a VTD reactor for scaled-up perovskite manufacturing because they omit the vapor deposition of the PbI_2_ film.

**Fig. 1:**
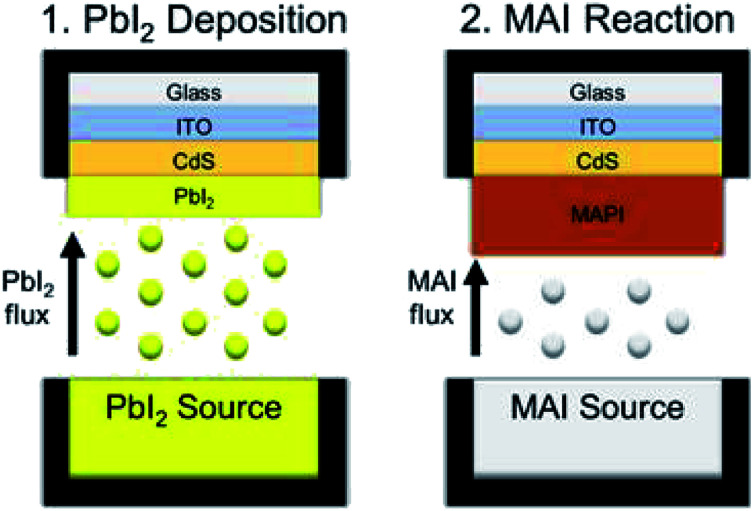
A schematic of the two-stage CSVT process used to deposit MAPI thin films.

The application of an all-vapor CSVT process to directly deposit methylammonium lead iodide (CH_3_NH_3_PbI_3_, MAPI) thin films must differ from CdTe deposition by CSVT, where the absorber sublimes from a single molecular source to form Cd and Te_2_ vapors that react at the substrate and form a solid film,^16^ because the vapor pressures of the constituent compounds of MAPI perovskites (PbI_2_ and MAI) are too dissimilar to deposit stoichiometric films from a single source.^20,21^ Therefore, a two-stage CSVT process has been developed to form the MAPI absorber layer by depositing a film of PbI_2_ and reacting it in MAI vapor as shown in Fig. 1. Here we report the deposition of PbI_2_ films *via* CSVT, their reaction with MAI vapor, a reaction kinetic model, an upper-threshold to reaction temperature, and functional devices achieving 12.1% efficiency.

## 2 Experimental approach

### 2.1 Substrate preparation

Soda-lime glass (SLG) substrates were sequentially cleaned by sonication in LiquiNOX® and CitraNOX® soaps, followed by rinsing in DI water and drying at 180 °F for 1 hour. After drying, a 300 nm layer of ITO was RF sputtered at room temperature onto the substrates through a shadow mask to define cell area.

### 2.2 CdS deposition by chemical surface deposition

CdS was chosen as the electron transport layer because its conduction band lies 0.3 eV below the perovskite's conduction band and due to its good hole-blocking properties.^22^ CdS was deposited on the ITO-coated SLG substrates using chemical surface deposition (CSD).^23^ The SLG/ITO substrates were heated on a hot plate to 55 °C and a solution was prepared containing 2.2 mL 1.5 mM CdSO_4_ (99.996%, Alfa Aesar), 2.2 mL 1.5 M thiourea (99%, Alfa Aesar), 2.8 mL 30% NH_4_OH (JT Baker) and 15 mL DI water. Afterward, 1.5 mL of the solution was dispensed dropwise onto each of the hot substrates. After 5 minutes, the substrates were removed from heat, tipped of fluid, rinsed with flowing DI water, and dried with argon. The process was repeated to apply a second coat of CdS, yielding a uniform, ≈50 nm thick layer.

### 2.3 CSVT reactor

**Fig. 2:**
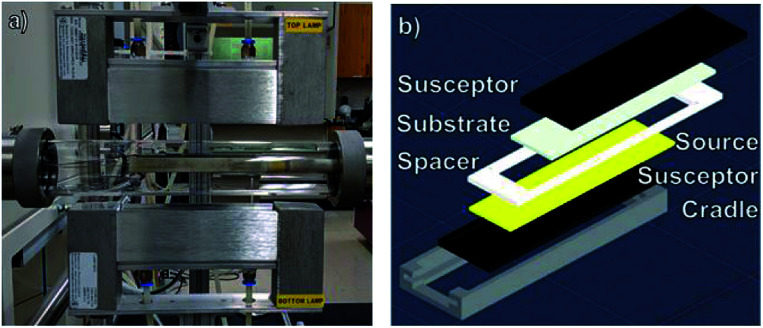
(a) A photograph of the CSVT reactor. (b) An exploded view of the CSVT fixture and its components.

A photograph of the CSVT reactor developed for these experiments is shown in Fig. 2a. It consists of a planar source-substrate geometry with the source positioned beneath the substrate. A motorized arm is used to position the apparatus in a 4.5 cm diameter quartz tube between two 1000 W lamps. These lamps independently heat two graphite susceptors, which are in direct contact with the source material and the substrates, as seen in Fig. 2b. Eurotherm 2404 controllers moderate the temperature of each susceptor using embedded thermocouples so that the heating lamps control the source and substrate temperatures between 25 and 500 °C. A roughing pump, an argon carrier gas flowing at 10 sccm, two Baratron capacitance manometers, and a throttle valve maintain the working pressure between 0.1 and 100 torr. The CSVT reactor is integrated with a multi-function glove box used to prepare, load, and unload the sources and substrates in a nitrogen atmosphere with O_2_ < 0.1 ppm and H_2_O < 5 ppm. This allows the perovskite layer to be processed start-to-finish without exposure to ambient air.

Fig. 2b shows an exploded schematic of the source and substrate holder. Insulating Macor® ceramic fittings hold the 10.8 cm long source and substrate susceptors in place and maintain a temperature gradient across a 2.2 mm gap defined by the spacer. This gradient promotes mass transport from the source to the substrates, resulting in deposition and film growth. The fittings also define the deposition area to be ≈20 cm^2^, which accommodates three 2.5 cm × 2.5 cm substrates per run.

CSVT processing of MAPI is shown schematically in Fig. 1. CdS-coated SLG/ITO substrates are loaded face down into the CSVT chamber over a PbI_2_ source, and the system is pumped to a base pressure of 10^−7^ torr. The chamber is then backfilled with argon gas to a fixed working pressure, and the system is allowed to equilibrate for 5 min. The lamps ramp the source and substrate susceptors to their temperature setpoints over 5 min at a fixed rate of 47 °C min^−1^ for the source and 38 °C min^−1^ for the substrate. The source and substrate temperatures are then controlled to within ±2 °C of their setpoints for a specified duration by independently modulating the top and bottom lamp power.

Following PbI_2_ deposition, the PbI_2_-coated substrates and the PbI_2_ source are unloaded from the CSVT reactor. Then a second source, filled with MAI powder, is loaded into the CSVT reactor with the PbI_2_-coated substrates. After pumping and backfilling, the source and the substrate susceptors are heated to their setpoints at a rate of 25 °C min^−1^. MAI sublimes and saturates the headspace surrounding the PbI_2_ film, driving a solid–vapor reaction to form MAPI. Finished MAPI films are removed from the CSVT chamber into the glove box, washed with isopropanol (anhydrous 99.5%, Sigma Aldrich) to remove residual MAI, blow-dried with argon, and annealed on a hot-plate at 100 °C for 5 minutes.

### 2.4 Rear contact preparation

Spiro-OMeTAD (Spiro) (99%, Sigma Aldrich) films were deposited using an established recipe from literature.^24^ The Spiro solution was stirred for 10 minutes and filtered through a 0.2 μm PTFE filter to remove large particles prior to spin-coating. Spiro films were dynamically spin-coated by aliquoting 100 μL of solution onto the substrate at 4000 rpm for 10 seconds. The films were dried and stored in the dark in a dry air desiccator (relative humidity < 7.5%) overnight prior to metallization.

Oxidized SLG/ITO/CdS/MAPI/Spiro samples were loaded into an electron beam evaporator and pumped to a base pressure of 10^−6^ torr. Gold films were deposited through a shadow mask at a rate of 6 Å s^−1^ to a final thickness of 100 nm. The active area of the solar cells was defined by the overlap area between the gold and the ITO to be 0.24 cm^2^. The edges of the perovskite were removed with a razor blade to expose the ITO contacts, and silver paste was applied using Dotite® D-550 Silver Colloid (2SPI).

### 2.5 Device measurement

Current–voltage (*J*–*V*) measurements were carried out using a setup maintained in the N_2_-filled glovebox. The intensity of a xenon arc lamp (Newport Oriel 67005 Housing; Newport Oriel 69907 Power Supply) was calibrated using a Si solar cell with a Schott KG-5 IR filter attached to the front. This limits the spectral sensitivity of the Si calibration cell to 800 nm and matches the spectral sensitivity of the calibration cell to test devices. The calibration value is based on quantum efficiency (QE) measurements and *J*–*V* measurements on a class A Oriel solar simulator.

*J*–*V* measurements were carried out in a 4-point probe configuration with scans between −0.4 and 1.2 V at a sweep rate of 200 V s^−1^ with 48 data points. Cells were scanned from reverse to forward bias then back. Afterward, cells were pre-biased at 1.2 V for 60 seconds prior to measurement from 1.2 V forward bias to −0.4 V reverse bias. The data exhibited minor oscillations due to capacitive coupling between the device and the source measure unit (SMU) or temporal variations in the light intensity, and, therefore, the data was smoothed using a 5-point adjacent average for clarity.

### 2.6 Materials characterization

Three scanning electron microscopes (SEM) were used to collect micrographs of various samples. An AMRAY 1810T Digital SEM was used at an accelerating voltage of 20 kV to image MAPI samples reacted at 160 °C and perform energy dispersive X-ray spectroscopy (EDS). A Zeiss Auriga 60 High Resolution Field Emission SEM and a JSM-7400 High Resolution SEM were used at 3 kV to image plan-view and cross-section imaging, respectively.

X-ray fluorescence (XRF) measurements were obtained using an Oxford Instruments X-Strata 980 Coating Thickness Analyser to measure the thickness of the PbI_2_ films. Samples were measured over a 0.75′′ × 0.875′′ area of the film using a 7 × 8 grid for a total of 56 equally-spaced data points that were used to determine the average thickness of each film.

The CSVT films were analyzed by X-ray diffraction (XRD) using a Philips/Norelco powder X-ray diffractometer using a CuKα X-ray source operating at 35 kV and 20 mA in Bragg–Brentano parafocusing geometry. The scan settings were 0.05° 2*θ* per step with a 4 second dwell time, over the 2*θ* range 10–30° to cover the principal peaks of PbI_2_ and MAPI. The XRD data was smoothed and processed with the Rachinger correction to remove contributions from CuKα_2_.^25^ All peaks were indexed and assigned to phases based on *d*-spacing conformity with references for ITO (cubic In_2_O_3_, ICDD 00-006-0416), PbI_2_ (hexagonal, ICDD 01-080-1000), and MAPI (tetragonal, pattern generated using PowderCell 2.0 for space group *I*4*cm*).

The weight percent of PbI_2_ (*w*_PbI_2__) in each sample was estimated from the intensity ratio (*R*) of the PbI_2_ (001) and MAPI (110) reflections, corrected for background, using standard quantitative powder diffraction analysis as shown in eqn (1).^26^ The detection limit of ≈0.1% is governed by the signal-to-noise of the data, which is governed by the scattering power of each phase, the instrumental noise, and the scan conditions.1
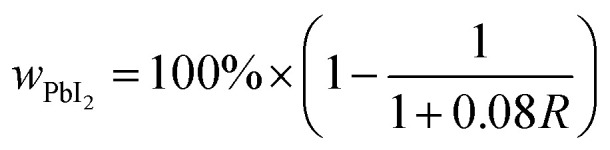


## 3 Results

### 3.1 Deposition of PbI_2_ thin films

Previous research indicates that for single-junction, perovskite solar cells, the optimal thickness of the MAPI absorber layer is ≈400 nm,^27^ which requires the PbI_2_ precursor film to be ≈200 nm thick.^28^ PbI_2_ was deposited using a source temperature (*T*_source_) = 260 °C, and a substrate temperature (*T*_sub_) = 215 °C, according to a Clausius–Clapeyron fit of its vapor pressure 
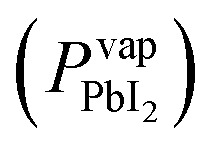
,^20^ to produce a vapor pressure difference of two orders of magnitude between the source (*P*^vap^_source_) = 2 × 10^−6^ ± 8 × 10^−7^ torr and the substrate (*P*^vap^_sub_) = 6 × 10^−8^ ± 3 × 10^−8^ torr. Depositions were carried out at a pressure (*P*) = 1 torr and the deposition time (*t*) was varied to achieve a desired film thickness. A 20 min deposition (*t* = 20 min) produced films with an average thickness of 225 ± 7 nm, measured by XRF.

**Fig. 3:**
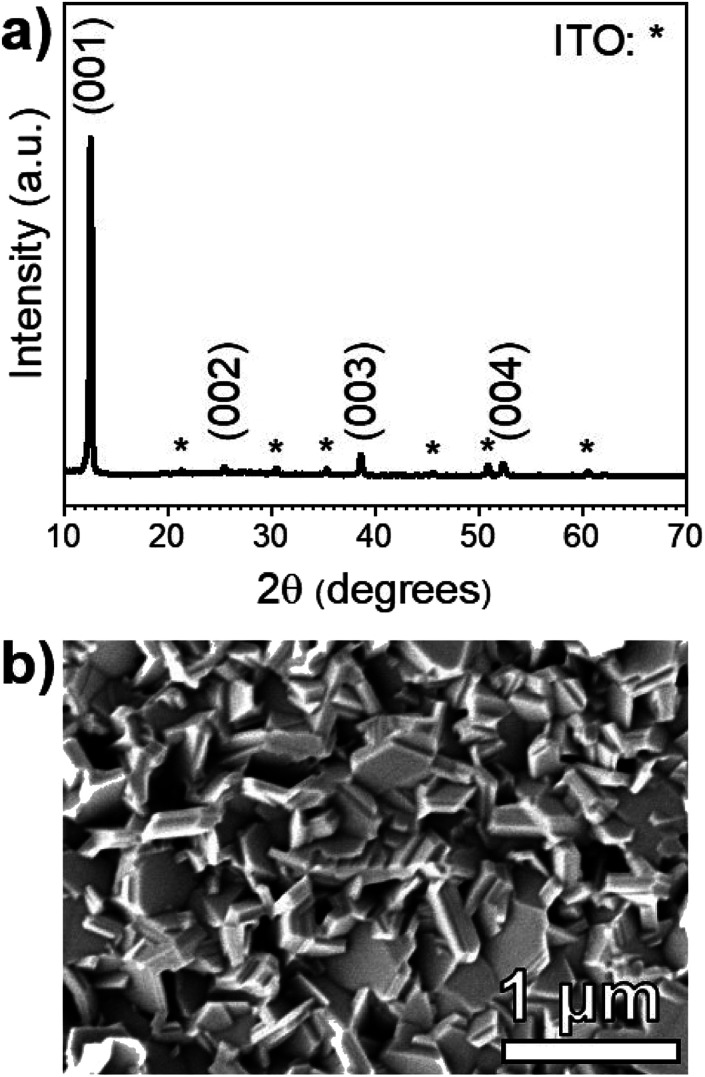
(a) XRD pattern of *a* ≈ 225 nm thick PbI_2_ film deposited on an SLG/ITO/CdS substrate at *T*_source_ = 260 °C, *T*_sub_ = 215 °C, *P* = 1 torr, and *t* = 20 min. The principal PbI_2_ planes are labeled at their respective diffraction peaks, and the ITO peaks are marked with an asterisk. (b) A plan-view SEM micrograph of the PbI_2_ film.

The XRD pattern of a PbI_2_ film on an SLG/ITO/CdS substrate shown in Fig. 3a is fully indexed using hexagonal PbI_2_ and cubic In_2_O_3_ standards. Notably, CdS diffraction peaks are not observed due its low thickness (≈50 nm) and poor crystallinity. The PbI_2_ XRD pattern shows the strongest diffraction peak at 2*θ* = 12.60°, which corresponds to the (001) basal plane of hexagonal PbI_2_. In a random pattern of PbI_2_ powder, the brightest peak occurs at 25.90° and corresponds to the (011) plane. This suggests that the PbI_2_ films deposited on CdS orient around the basal plane during the deposition process.

Fig. 3b shows a plan-view SEM micrograph of the same PbI_2_ film. The morphology consists of hexagonal platelets that are consistent with the quasi-two-dimensional, 2H polytype of the PbI_2_ hexagonal crystal structure.^29^ While the film appears to have a rough texture, the PbI_2_ platelets seem to fully coat the CdS substrate with no visible pinholes.

### 3.2 Reaction of PbI_2_ in MAI vapor

MAI reactions of PbI_2_ films were carried out isothermally at *T*_source_ = *T*_sub_ = 100 °C. The amount of MAI participating in the reaction is estimated from a Clausius–Clapeyron fit of the vapor pressure,^21^ which gives *P*^vap^_MAI_ = 50 ± 20 mTorr at *T* = 100 °C. The system pressure was maintained at a constant *P* = 9 torr using an argon ambient, which diluted the concentration of MAI vapor.

**Fig. 4:**
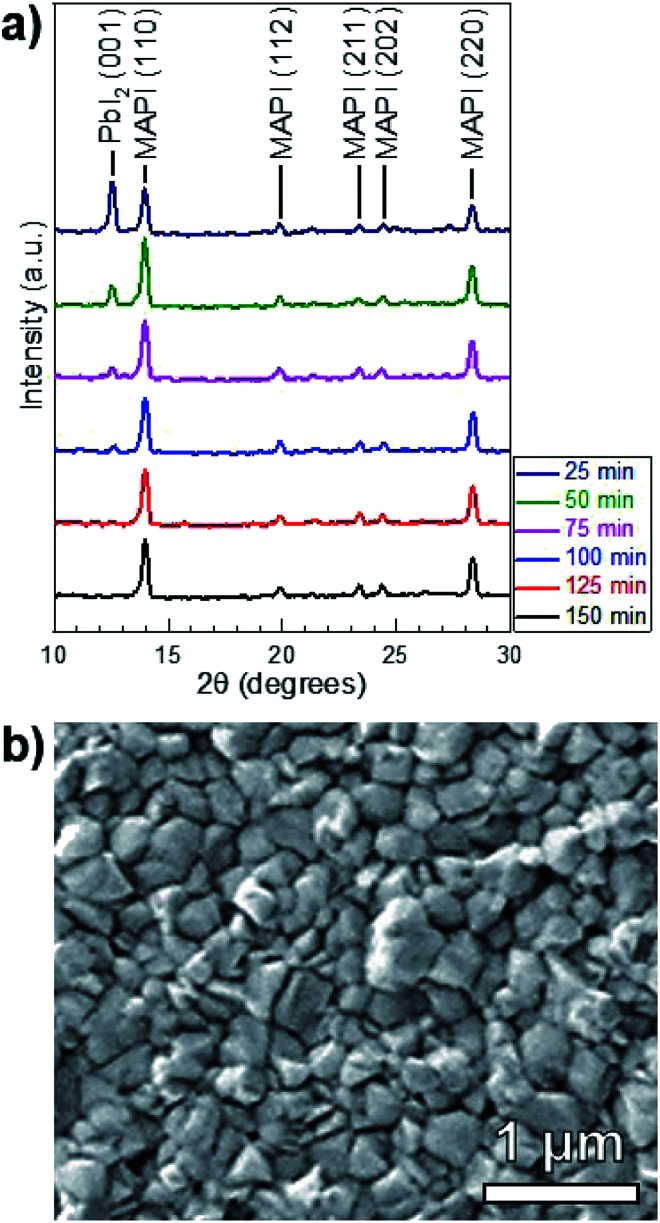
(a) XRD patterns of MAPI films reacted isothermally in MAI at *T* = 100 °C and *P* = 9 torr for durations *t* = 25–150 min. The principal PbI_2_ and tetragonal MAPI peaks (space group *I*4*cm*) are labeled. (b) A plan-view SEM micrograph of the MAPI film reacted for 150 min.

A series of reactions were carried out from 25–150 min in 25 min increments. Fig. 4a shows the XRD patterns of the reacted films at each point in time. The conversion of PbI_2_ to MAPI is confirmed by the disappearance of the PbI_2_ (001) diffraction peak at 12.60° and the appearance of the MAPI (110) and (220) peaks at 14.05° and 28.40° respectively. The PbI_2_ (001) intensity decreases and the MAPI (110) signal increases with increasing reaction time. After a 125 min reaction, the PbI_2_ signal is indistinguishable from the background. The morphology of the 150 min-reacted film from Fig. 4a is shown in Fig. 4b. The film has a dense morphology that appears to be continuous across the CdS substrate without visible pinholes.

**Fig. 5:**
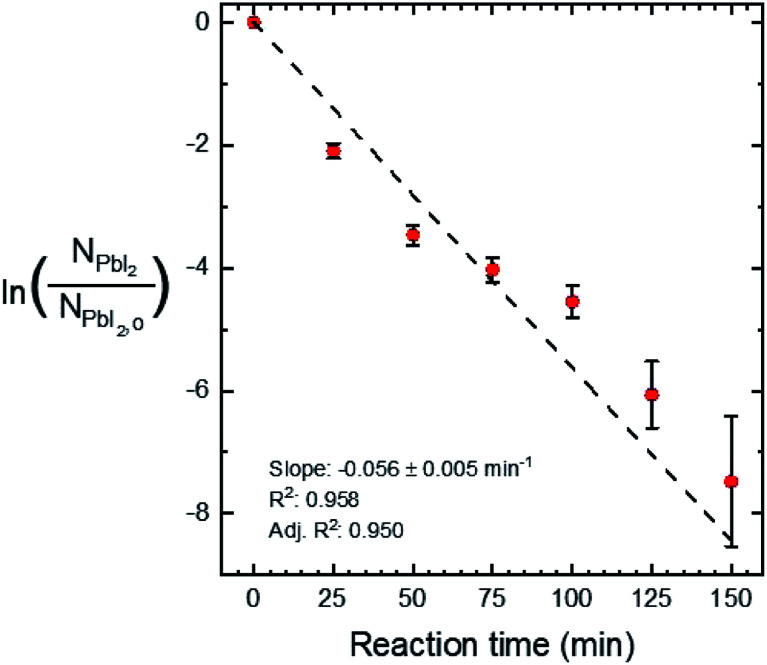
The natural log of *N*_PbI_2__ normalized to *N*_PbI_2_,o_*versus* reaction time. The error bars show 95% confidence intervals with increasing uncertainty with increasing reaction time, due to a decreasing signal-to-noise ratio in the XRD measurement. A linear fit of the data is shown along with its slope, *R*^2^, and adjusted *R*^2^.

Quantitative X-ray diffraction intensity analysis^26^ was performed on the XRD patterns shown in Fig. 4a. A comparison of the PbI_2_ (001) and MAPI (110) peak intensities yielded the weight percent of PbI_2_ as a function of the reaction time, which was converted to moles (*N*_PbI_2__) and plotted in Fig. 5. As *t* increases, *N*_PbI_2__ decreases as it is converted to MAPI. This shows >99% conversion from PbI_2_ to MAPI after *t* = 125 min at *T* = 100 °C and *P* = 9 torr.

A linear fit of the data in Fig. 5 is used to approximate the first-order kinetic expression in eqn (2), where *k* is the rate constant and *t* is reaction time.2
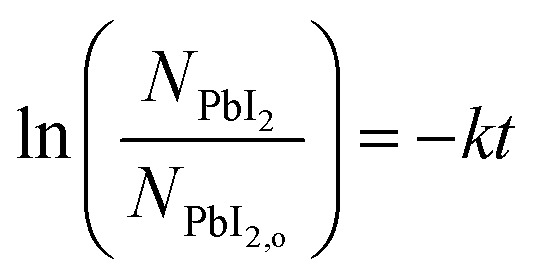


The associated *R*^2^-value of 0.958, and an adjusted-*R*^2^-value of 0.950, indicate that the first-order kinetic model is in reasonable agreement with the data; however, there is some visual evidence of curvature in the data, which may indicate a slightly more complicated functional relation. Consequently, we estimate the reaction rate constant, *k*, from the slope of the line as *k* = 0.056 ± 0.005 min^−1^. While this rate parameter is low, leading to reactions requiring 150 min to reach >99% completion, it is likely to be a function of the reaction temperature and the concentration of MAI vapor, which are not explicitly included in the first-order model of eqn (2). This suggests that the reaction temperature and pressure can be tuned for faster reactions.

**Fig. 6:**
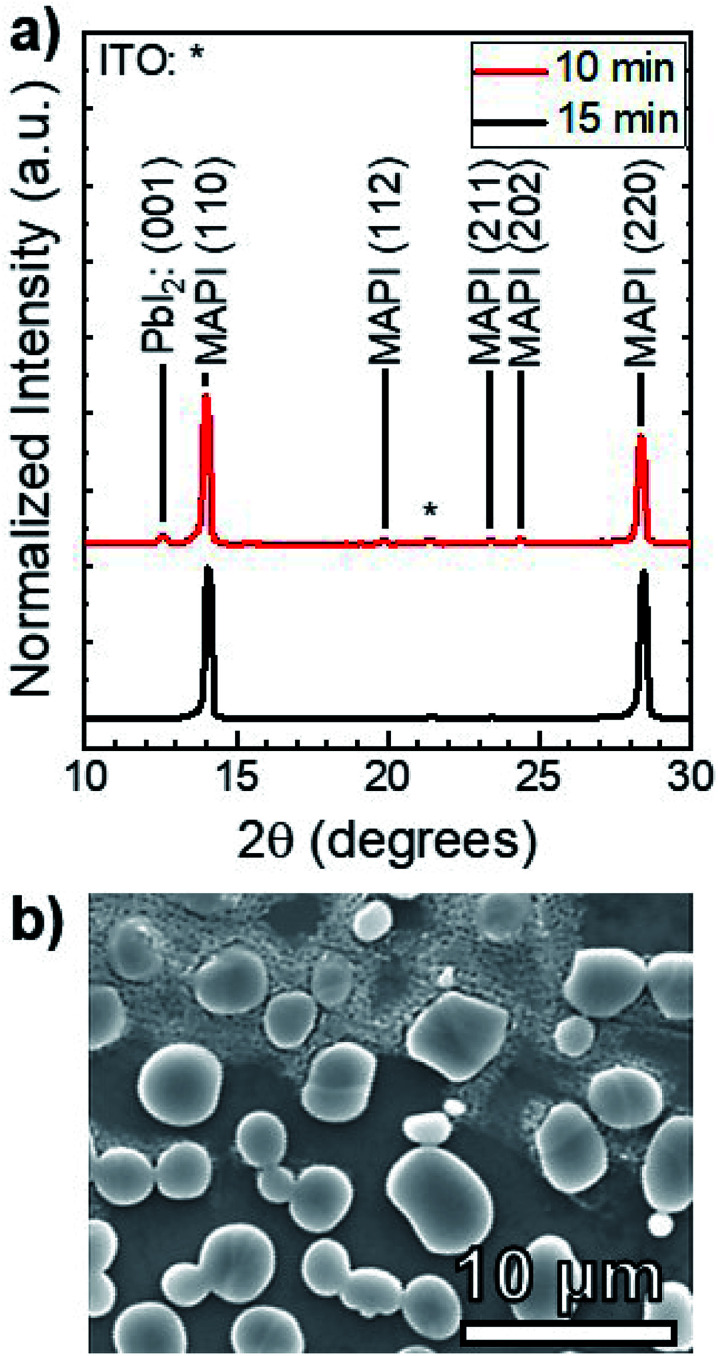
(a) XRD patterns of MAPI films reacted isothermally at *T* = 160 °C and *P* = 9 torr with durations of *t* = 10 min and *t* = 15 min. The PbI_2_ (001) peak and the tetragonal MAPI peaks are labeled. (b) A plan-view SEM micrograph of the 15 min-reacted MAPI film.

To investigate the effect of increased temperature and MAI concentration, another set of MAI reactions were carried out at *T* = 160 °C and *P* = 9 torr, corresponding to an MAI vapor pressure of *P*^vap^_MAI_ = 6 ± 1 torr^21^—approximately two orders of magnitude higher than that of the 100 °C reactions. Fig. 6a shows the XRD patterns of films after 10 and 15 min reactions. There is a distinct PbI_2_ (001) peak at 12.60° for the 10 min reaction, whereas the PbI_2_ peak is indistinguishable from the background after 15 min. According to our quantitative analysis, this indicates >99% conversion from PbI_2_ to MAPI. This reaction at *T* = 160 °C is an order of magnitude faster than the reaction at *T* = 100 °C—indicating the rate of reaction is, in fact, a function of temperature. It is worth noting that the MAPI (112), (211), and (202) peaks are present in both the 10 and 15 min reactions.

Fig. 6b shows that the resulting MAPI films have a discontinuous morphology of large agglomerates on the substrate surface. EDS was used to characterize the films and confirmed that the light gray agglomerates were MAPI and the dark background was the SLG/ITO/CdS substrate. This agglomeration may be caused by de-wetting from the substrate surface during the reaction process—possibly due to high interfacial surface energy between the substrate and the perovskite. Alternatively, it may be due to Ostwald ripening where small, thermodynamically unstable particles are incorporated into larger, more stables particles to minimize interfacial surface energy.^30^ Both phenomena typically occur at elevated temperatures due to increased adatom mobility,^31^ and suggest that the 160 °C reaction temperature is too high for MAPI processing on CdS at *P* = 9 torr.

### 3.3 Characterization of solar cells

**Fig. 7:**
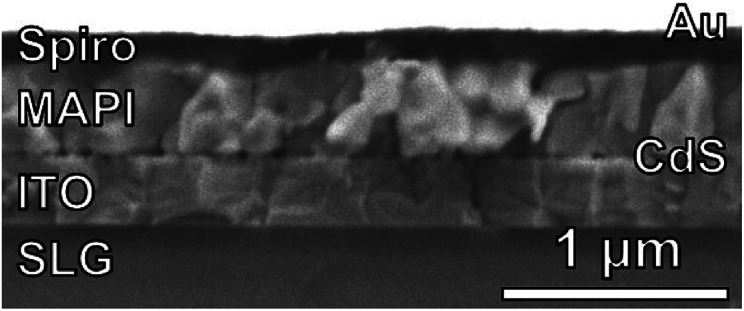
A cross-sectional SEM image of a completed device with an SLG/ITO/CdS/MAPI/Spiro/Au stack.

Fully-reacted MAPI films from 150 min reactions at 100 °C were integrated into functional solar cells with an SLG/ITO/CdS/MAPI/Spiro/Au structure as shown in the cross-sectional SEM image of Fig. 7. The MAPI and Spiro layers appear to form pinhole-free films with only small thickness variations due to the intrinsic roughness of the MAPI layer. The MAPI grains appear to be dense and columnar with a grain width of ≈150–200 nm. Furthermore, the reaction appears to have doubled the thickness of the original PbI_2_ film from ≈225 nm of PbI_2_ to ≈450 nm of MAPI.

**Fig. 8:**
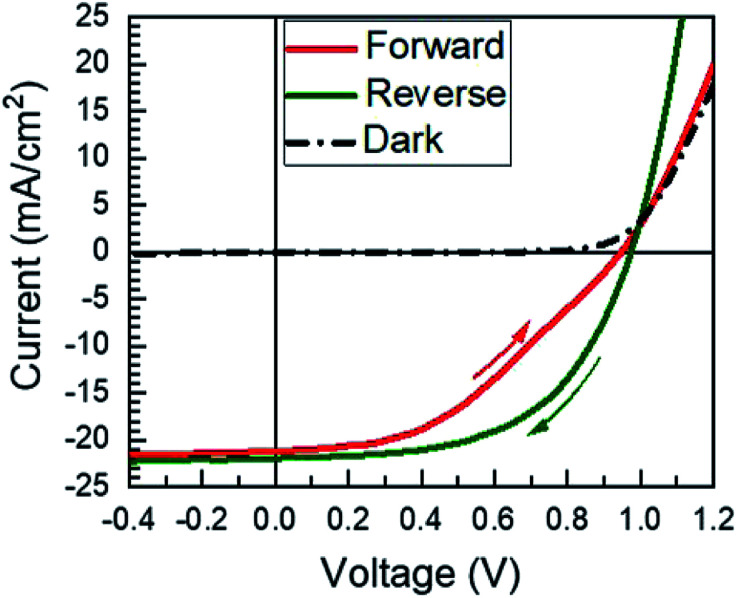
The *JV* curve of the champion solar cell presented in this work. The arrows indicate the direction of the scan.

A total of 50 solar cells were fabricated with identical PbI_2_ deposition, MAI reaction, and device processing conditions. A champion cell performance of 12.1% efficiency was achieved on a reverse sweep with open-circuit voltage (*V*_OC_) = 980 mV, short-circuit current (*J*_SC_) = 21.9 mA cm^−2^, and fill factor (FF) = 56.6% as shown in Fig. 8. However, this device exhibited hysteresis as evidenced by a lower efficiency of 8.4% during the forward sweep. This lower efficiency is due primarily to a loss in FF with *V*_OC_ = 950 mV, *J*_SC_ = 21.1 mA cm^−2^, and FF = 41.9%. The higher performance in the reverse sweep is consistent with previous reports, which have attributed hysteretic effects to ion migration within the MAPI layer, where a positive voltage bias leads to better band alignment and improved carrier collection.^32^

The compiled cell parameters for all 50 devices are shown in Table 1. On average these cells exhibit a reasonable *V*_OC_ and *J*_SC_ of 968 mV and 18.3 mA cm^−2^ that can be incrementally improved upon to achieve high efficiency solar cells. However, there are substantial losses in FF that limit the efficiency. These losses can be attributed primarily to the CdS electron transport layer because, in addition to parasitic absorption and a small conduction band spike,^33^ it has been reported that CdS can interact with excess MAI to form an interfacial Cd perovskite (MA_2_CdI_4_).^34^ This would create a blocking barrier and reduce both the *J*_SC_ and the FF.^34^ Therefore, it is likely that replacing CdS with a more appropriate electron transport layer, such as SnO_2_ or TiO_2_, could be used to improve the FF and the overall cell efficiency.

**Table 1:** Device results and statistics for solar cells with a sample size of *n* = 50. The error represents the 95% confidence interval around the average for each parameter

Parameter	Average	Standard deviation
*V*_OC_ (mV)	968 ± 6	20
*J*_SC_ (mA cm^−2^)	18.3 ± 0.6	2.1
FF (%)	47.4 ± 1.4	5.0
PCE (%)	8.4 ± 0.3	1.1

## 4 Discussion

This all-vapor CSVT process serves as an advance toward developing a commercial vapor deposition method for the production of perovskite thin films. However, there are several concepts that must be better understood before designing and implementing scaled VTD reactors. First, the perovskite field has moved away from the CH_3_NH_3_PbI_3_ composition toward alloys that include formamidinium, Cs, and Br because they have proven to be more stable and more efficient.^3^ It is apparent that a commercial perovskite production process will require the flexibility and methods to incorporate alloys into the material. Second, a quantitative mass transport model of the vapor deposition process is necessary to develop a scaled-up process. The composition of the alloyed perovskite film is essential to its performance and stability; therefore, a model is needed to guide experimentation toward a targeted composition. Finally, VTD of perovskites could be carried out either *via* a sequential process, as illustrated through CSVT, or *via* a simultaneous process. While the sequential process is limited by the rate of PbI_2_ conversion, a sequential process could circumvent this issue by co-depositing the perovskite's constituent materials through a low-vacuum and high-throughput process. Any effort to address one of these outstanding issues would be of great importance in developing perovskite vapor processing at the commercial scale.

## 5 Conclusions

This work establishes a sequential, all-vapor CSVT process to fabricate single-phase CH_3_NH_3_PbI_3_ perovskite thin films. Vapor-processed PbI_2_ films deposited on a CdS substrate possess a hexagonal, platelet-like morphology oriented about the (001) basal plane. Reacting these films at 100 °C and 9 torr produces continuous MAPI films, but this reaction temperature yields a slow reaction that requires at least 125 min to reach complete conversion. The change in PbI_2_ phase content *versus* time was used to quantify the rate of reaction, however further study is needed to develop a more accurate kinetic mechanism. Reacting the PbI_2_ films at 160 °C and 9 torr yields complete conversion to MAPI in 15 min, but this elevated reaction temperature promotes de-wetting from the substrate which inhibits the formation of a continuous MAPI film. Devices fabricated from MAPI processed at *T* = 100 °C in the configuration SLG/ITO/CdS/MAPI/Spiro/Au achieved a champion cell efficiency of 12.1% with *V*_OC_ = 980 mV, *J*_SC_ = 21.9 mA cm^−2^, and FF = 56.6%. These results validate CSVT as a viable processing technique for perovskite solar applications and establish a foundation for further research into scaling perovskite production through VTD.

## Conflicts of interest

There are no conflicts to declare.

## Supplementary Material
